# Supplementary material for: Confinement of Skyrmions in Nanoscale FeGe Device-like Structures
Source: ACS Appl Electron Mater. 2022 Sep 7;4(9):4427–37. doi: 10.1021/acsaelm.2c00692 (PMC9520970; doi:10.1021/acsaelm.2c00692)
Supplement: Supplementary file 1 — el2c00692_si_001.pdf [file el2c00692_si_001.pdf]

Supporting Information
Confinement of Skyrmions in Nanoscale FeGe Device-like Structures

Alison C. Twitchett-Harrison,*,† James C. Loudon,† Ryan A. Pepper,‡ Max T. Birch,¶,§ Hans Fangohr,‡,∥ Paul A. Midgley,† Geetha Balakrishnan,⊥ and Peter D. Hatton¶

†*Department of Materials Science and Metallurgy, University of Cambridge, 27 Charles Babbage Road, Cambridge, CB3 0FS, United Kingdom.*

‡*Faculty of Engineering and Physical Sciences, University of Southampton, Southampton, SO17 1BJ, United Kingdom.*

¶*Department of Physics, Durham University, Durham, DH1 3LE, United Kingdom.*

§*Max Planck Institute for Intelligent Systems, 70569 Stuttgart, Germany.*

∥*Max Planck Institute for Structure and Dynamics of Matter, Luruper Chaussee 149, 22761 Hamburg, Germany.*

⊥*Department of Physics, University of Warwick, Coventry, CV4 7AL, United Kingdom.*

E-mail: act27@cam.ac.uk

1 Details of sample synthesis and preparation

The FeGe single crystals were grown by the chemical vapour transport method with iodine as the transport agent.[1] Magnetometry measurements were performed to characterise the bulk single crystal FeGe sample. The Curie temperature of the sample T_C, defined as the

point of greatest slope in a plot of the magnetisation M versus temperature, was found to be 280.5 K.

Figure S1 illustrates the stages used to prepare the device-like structures (as described in Materials and Methods). The dumbbell structures were prepared so that the central constriction was the width of a single skyrmion. Surrounding the device-like structures a dark band is visible at the edges between the FeGe structures and Pt coating that is likely to be a damaged surface layer created by the implantation of Ga^+ ions during sample preparation. This is approximately 15 nm in depth at each surface, estimated by measuring the thickness of the dark bands. The central constrictions were measured to be 75, 77 and 64 $\pm$ 3 nm in the three device-like shapes, comparable to the helical length of 70 nm in FeGe. The device-like structures were created to limit any skyrmion lattice that does form to $2-3$ skyrmions by $4-5$ skyrmions. Two simple blocks were also prepared with similar dimensions to allow a comparison of skyrmion behaviour in a less complex structure under the same experimental conditions. The plane of the sample lies close to $(01\bar{1})$ with the long axis of the sample corresponding to [111] and the short axis corresponding to $[2\bar{1}\bar{1}]$. The thickness of the device-like structures was determined using energy filtered imaging to be 73 $\pm$ 6 nm.[2]

2 Electron Microscopy

The thinned cross-section of FeGe device-like structures was mounted in a Gatan liquid nitrogen cooled model 636 transmission electron microscope (TEM) holder and examined in an FEI Titan[3] TEM equipped with a Lorentz lens. The specimen was initially mounted in a magnetic field-free condition and a calibrated external magnetic field was applied out of the plane of the specimen using the objective lens of the TEM. To observe magnetic contrast in the TEM, a phase imaging technique is required, and these are only sensitive to the in-plane components of the magnetic flux density arising from local magnetisation within the

specimen. Bloch skyrmions appear as bright or dark areas of contrast when imaged away from the focus depending on defocus and orientation of applied magnetic field. The helical phase can be characterised using defocused imaging, but the field polarised and cone phases cannot be distinguished from each other as there is no net in-plane component of magnetic field for either when an out-of-plane magnetic field is applied. Lorentz TEM (LTEM) image series were acquired at 90 K and 263 K with an applied external magnetic field varying between 0 mT and ±310 mT. Hysteresis experiments were conducted at 90 K, 219 K and 245 K and were imaged using LTEM. Images were energy filtered using a 10 eV Gatan Tridiem imaging filter and were acquired on a 2048 × 2048 pixel CCD. At each change of magnetic field within the hysteresis loop, the LTEM image was refocused before adjusting the defocus to 200 μm for each image acquired. The changes in applied magnetic field also give rise to subtle tilts of the electron beam causing diffraction contrast to move across the image so that some of the shapes appear dark in some of the images.

3 FIB damage of specimen surfaces

Focused ion beam milling is inherently damaging to the specimen surfaces, leaving both an amorphous layer and an ion implanted layer deeper into the specimen.[3] The specimen preparation method for these small device-like structures allows us to examine some of the surface layers in cross-section and explore the impact of these surface layers on the magnetic spin structures that are experimentally observed. Bright-field TEM images reveal a dark band at the surface of the FeGe nanostructures where surface modification has occurred due to sample preparation in the FIB. Intensity line traces were taken across each edge as shown in Figure S2(a) and (b) and the surface layer thickness was measured from the observed reduction in intensity as shown in Figure S2(b). The measured dark surface layer thicknesses are recorded in Figure S2 and vary around the edges of the device-like structures with the narrowest layers observed on the inner surfaces that are adjacent to

another nanostructure (i.e. surfaces 3 and 6). These are subjected to the lowest dose of ions compared to the other surfaces (1, 2, 4 and 5) where more material has been removed during the sample preparation process. Electron beam Pt deposition has occurred on all surfaces but this is not expected to have a significant impact on the thickness of the surface damage layers.[4] Using TRIM simulations[5] we can estimate that for a 30 kV Ga ion beam at 89.9° incident angle to the FeGe surface, 90 % of the ions are stopped within 8 nm of the surface. Recent analysis of the surfaces of a 30 kV Ga^+ ion FIB-prepared FeGe needle by Wolf *et al.*[6] has revealed that only the outermost 4 nm of the surface is amorphous, and that a strong signal from implanted gallium is observed using STEM-EDX to a depth of at least 17 nm into the surface, which would correspond to the observed thickness of the dark surface ('damage') layers observed in these specimens, particularly on the surfaces with lowest damage (3 and 6). When considering the magnetic properties at the surfaces of the specimen, we can observe that the helical phase magnetic contrast is observed to continue into the dark layer specimen. LTEM does not allow a detailed analysis of the magnetic contrast very close to the surfaces because overlapping contrast is observed at the edges from mean inner potential differences between the FeGe and Pt layer.

4 Mask generation for Micromagnetic Simulations

In order to construct the specimen geometry in the simulations, we start from a standard bright-field LTEM image, taken close to the image plane where little magnetic contrast is observed which allows the boundary between the Pt and FeGe to be clearly defined (see Figure S9(a)). A gaussian blur is used (Figure S9(b)) before an adaptive local histogram equalisation is applied to each image (Figure S9(c)), and intensity thresholding is then used to resolve a clear boundary (Figure S9(d) and (e)) which can be used to construct a mask defining the simulation geometry, using the software package scikit-image[7] (Figure S9(f)). By pipelining the processing of the experimental images in this way, we ensure that there is

consistency between the mask creation for each geometry.

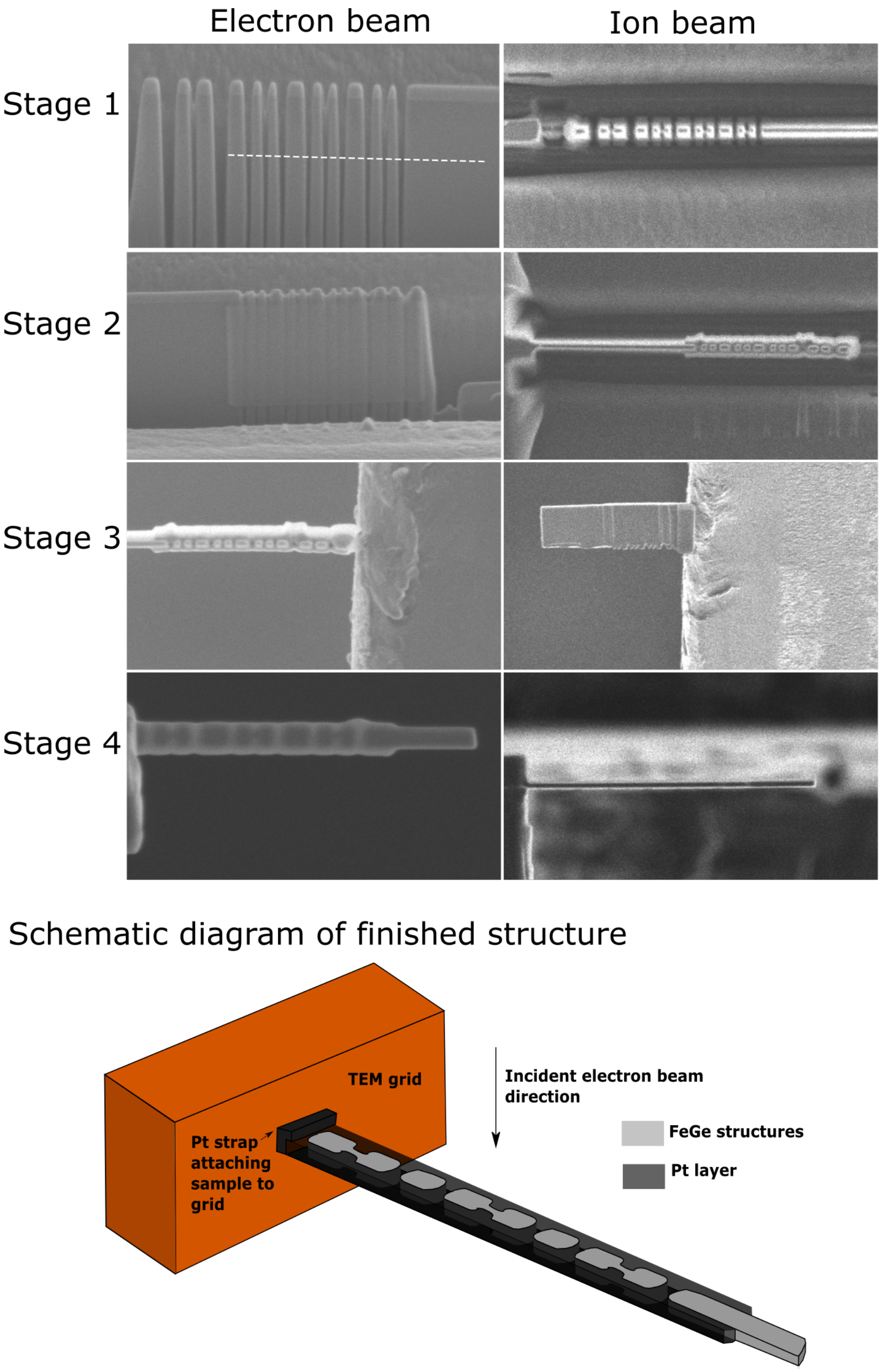

Figure S1: Secondary electron images acquired during specimen preparation. The ion beam is used in stage 1 to create the dumbbell and block shapes as tall FeGe pillars. The dashed white line indicates the position and length of the final TEM membrane. Electron beam platinum deposition is used in stage 2 to coat the pillars on both faces. The Pt-coated structures are lifted-out in stage 3 and attached to a TEM grid, and in stage 4 the pillars are thinned to create an electron transparent specimen containing the dumbbells and blocks.

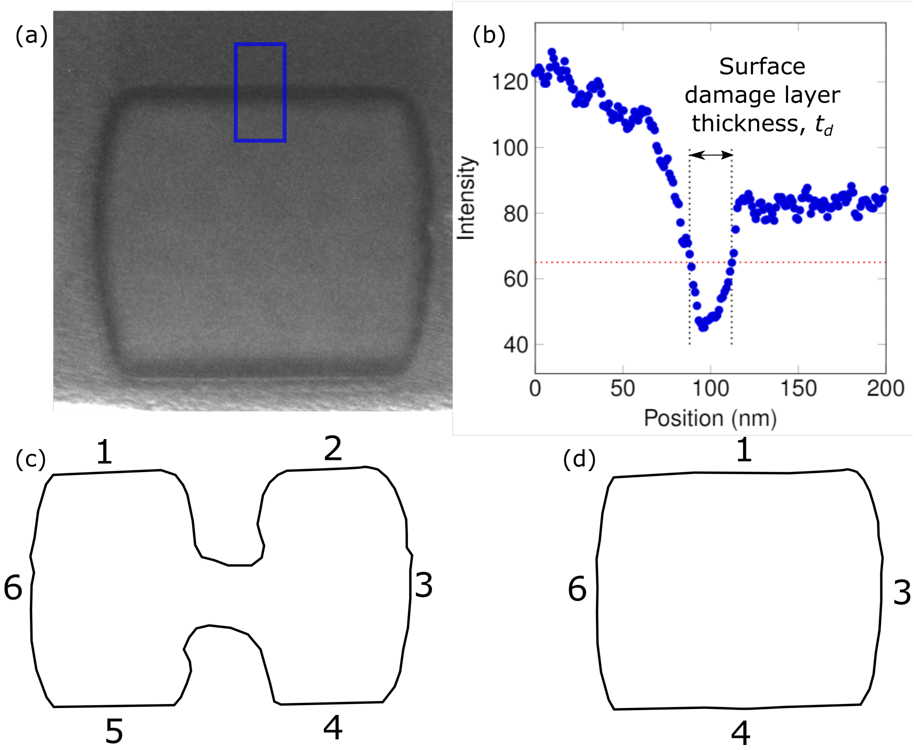

Position	Surface damage layer thickness, t_d (nm)
1	29.9 ± 1.3
2	30.0 ± 1.2
3	16.2 ± 4.2
4	37.0 ± 1.1
5	35.4 ± 1.2
6	23.9 ± 3.7

Shape	Perimeter (nm)	Area (nm^2)
D1	2430	191,000
B1	1660	167,000
D2	2440	197,000
B2	1580	155,000
D3	2390	186,000

Figure S2: (a) Bright field image of B2. The blue rectangle marks the area from which an intensity line scan (projected along the edge) was taken, shown in (b). Numbered positions around the edges of the (c) dumbbell and (d) block structures. Table of measured average surface damage layer thickness at each position. Table of shape with measured perimeter and area (to the nearest 1000 nm^2).

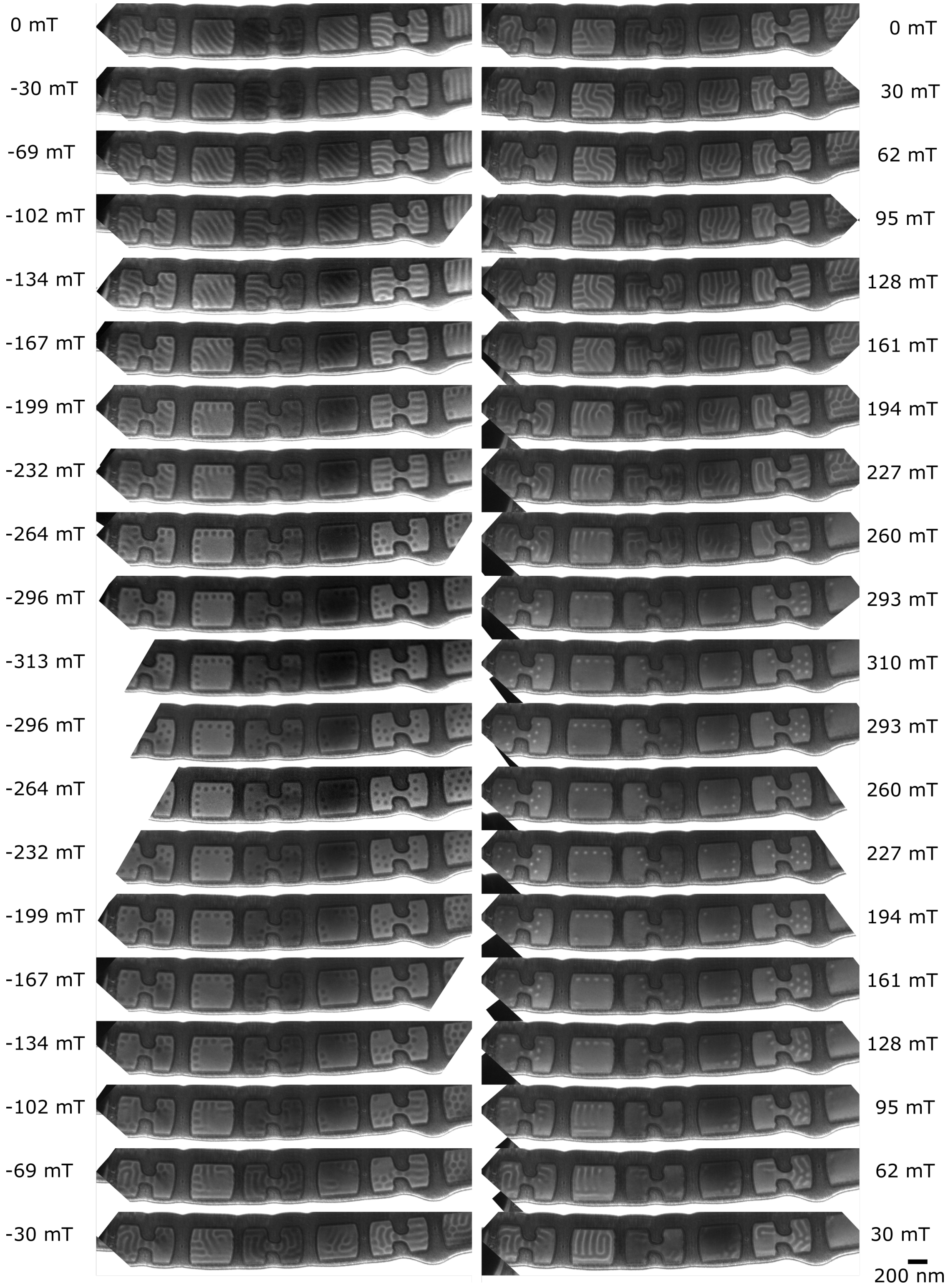

Figure S3: Unprocessed LTEM images of the device-like structures at 90 K under an applied external magnetic field cycled from 0 mT to −313 mT and then to +310 mT before returning to 0 mT.

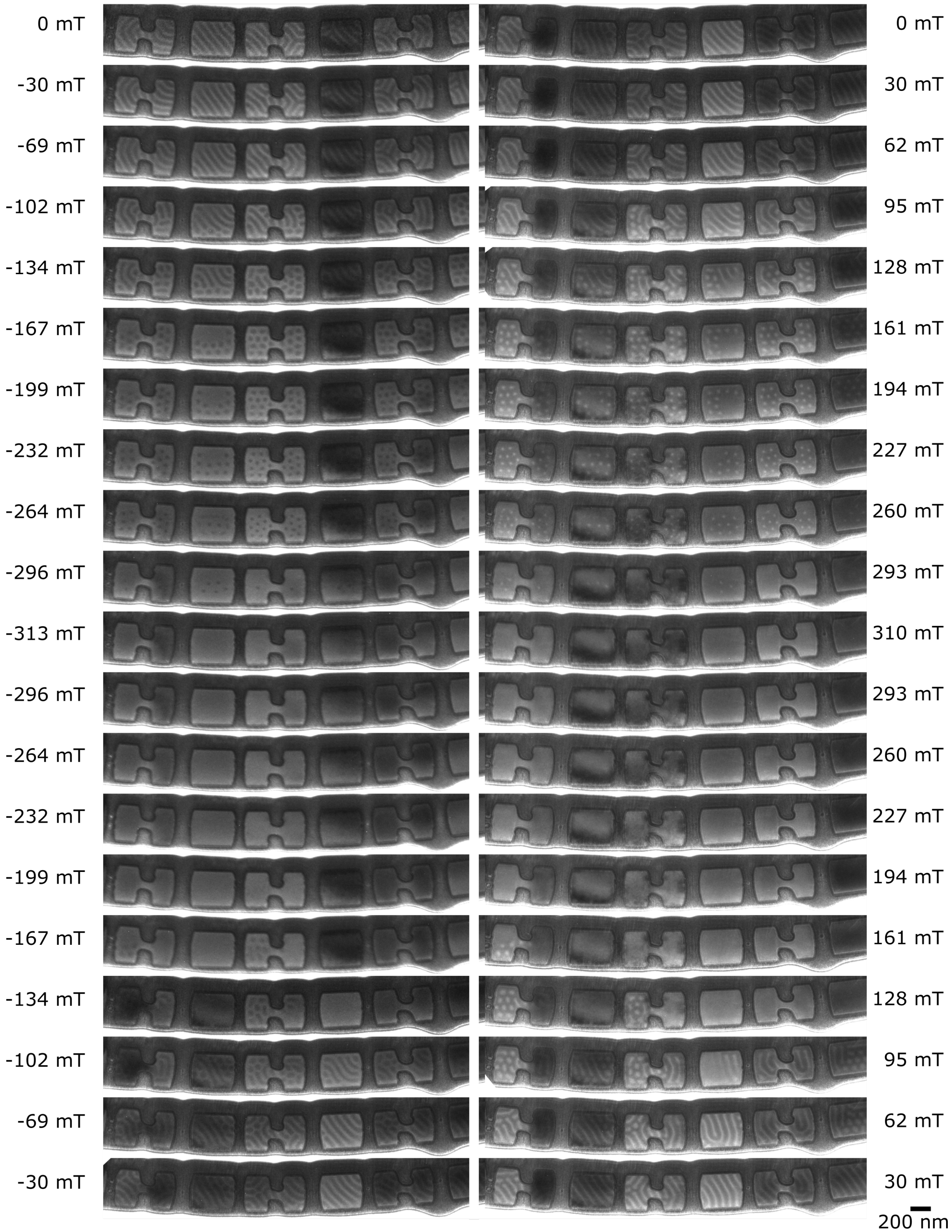

Figure S4: Unprocessed LTEM images of the device-like structures at 219 K under an applied external magnetic field cycled from 0 mT to −313 mT and then to +310 mT before returning to 0 mT.

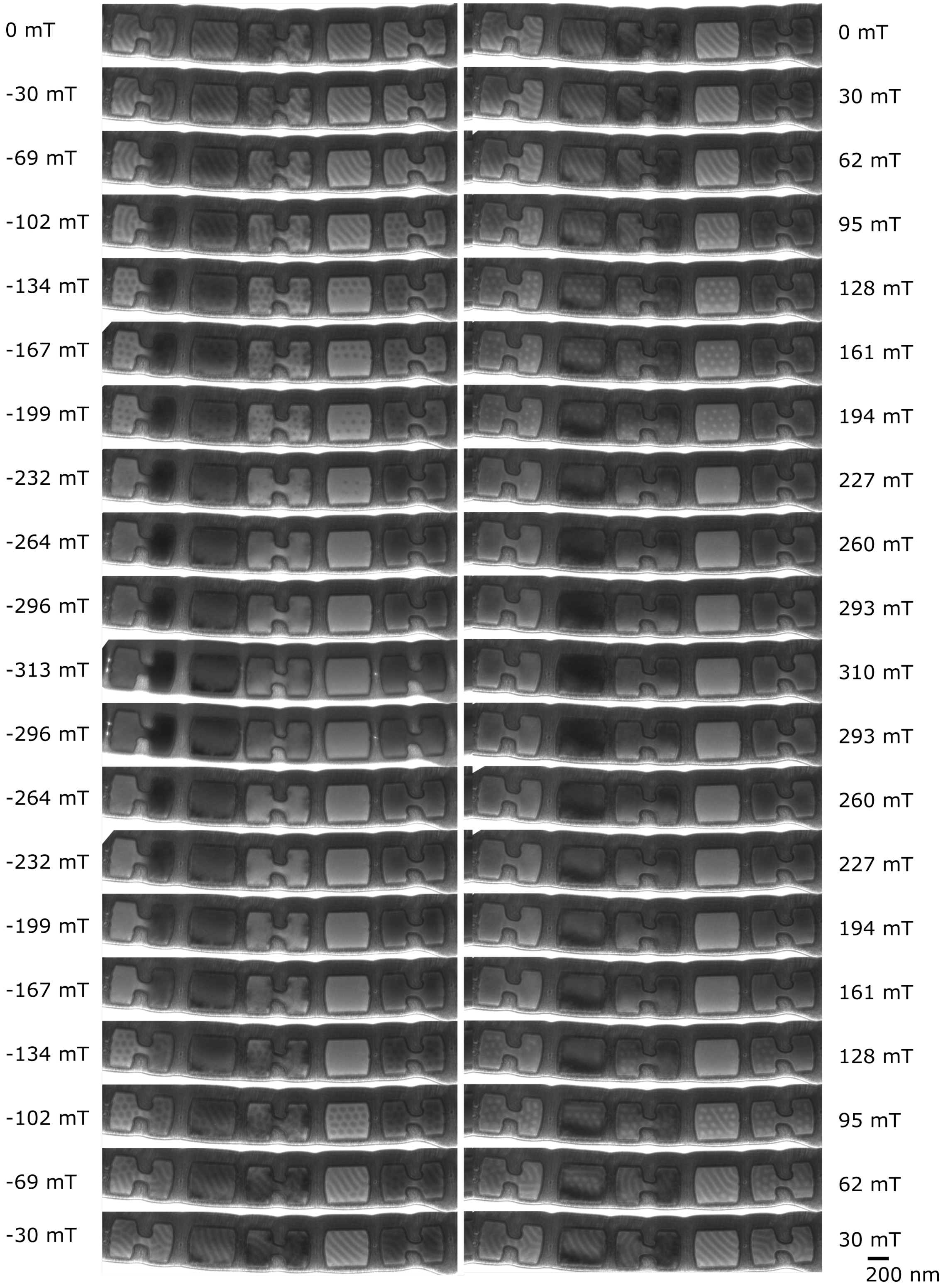

Figure S5: Unprocessed LTEM images of the device-like structures at 245 K under an applied external magnetic field cycled from 0 mT to −313 mT and then to +310 mT before returning to 0 mT.

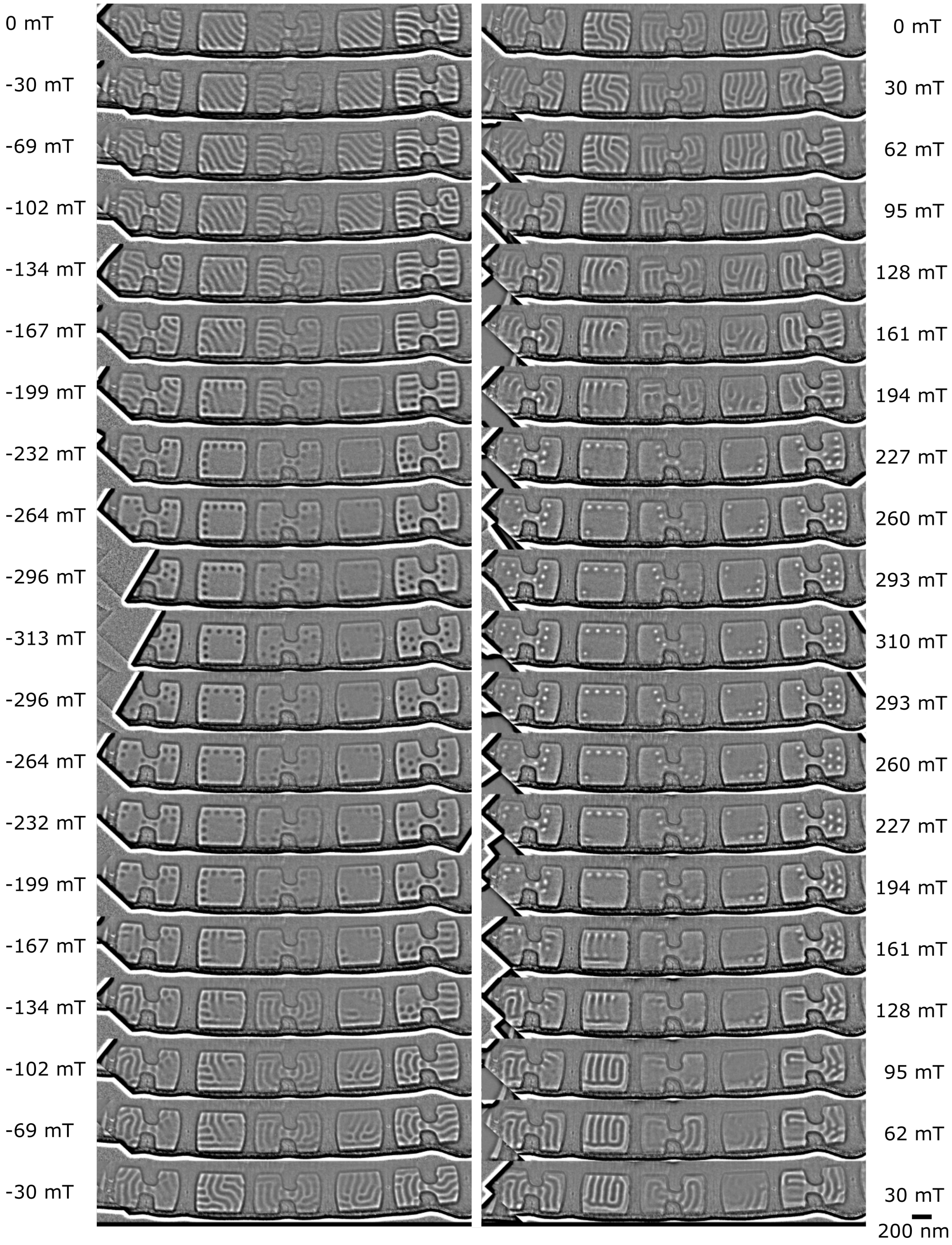

Figure S6: High-pass filtered LTEM images of the device-like structures at 90 K under an applied external magnetic field cycled from 0 mT to −313 mT and then to +310 mT before returning to 0 mT.

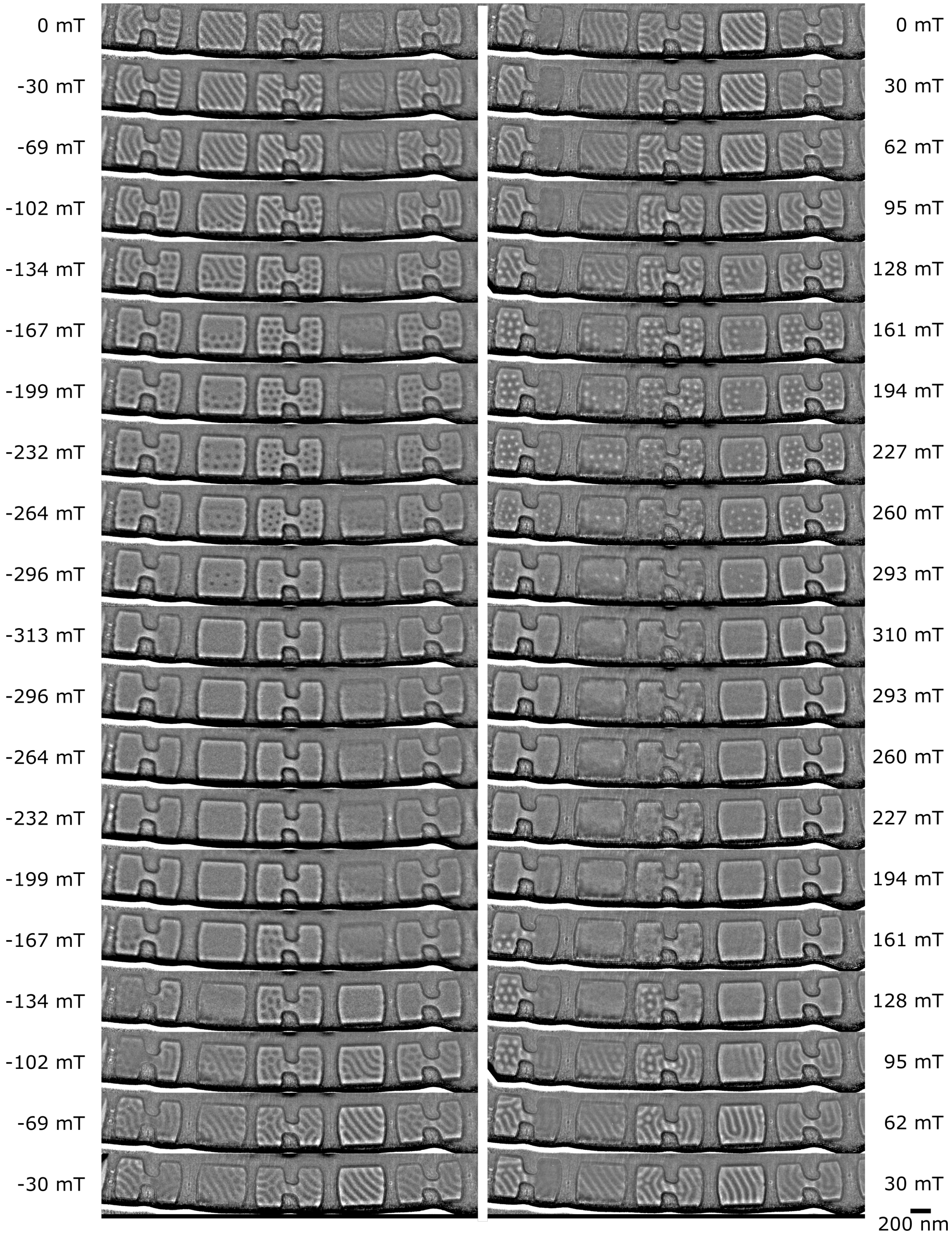

Figure S7: High-pass filtered LTEM images of the device-like structures at 219 K under an applied external magnetic field cycled from 0 mT to −313 mT and then to +310 mT before returning to 0 mT.

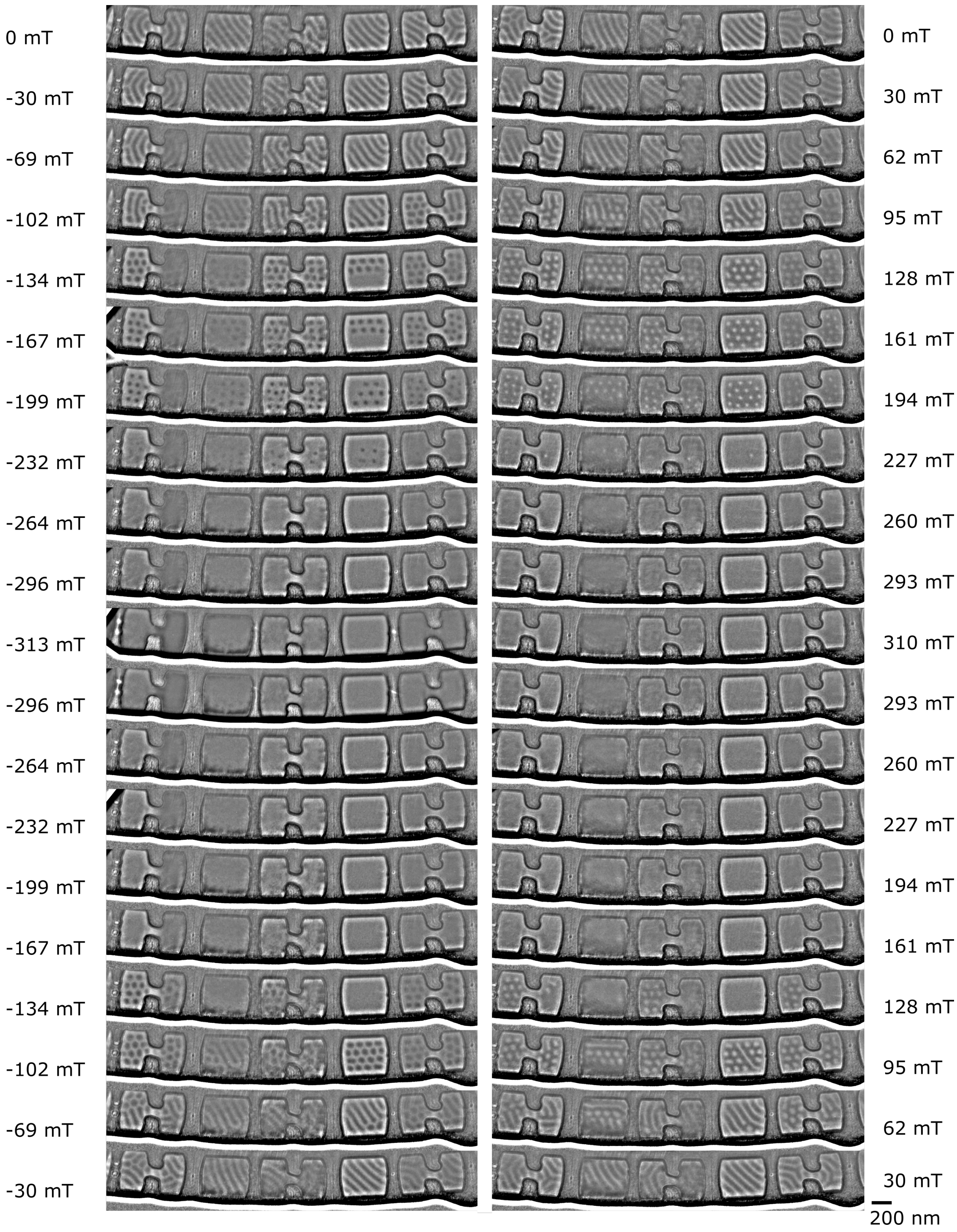

Figure S8: High-pass filtered LTEM images of the device-like structures at 245 K under an applied external magnetic field cycled from 0 mT to −313 mT and then to +310 mT before returning to 0 mT.

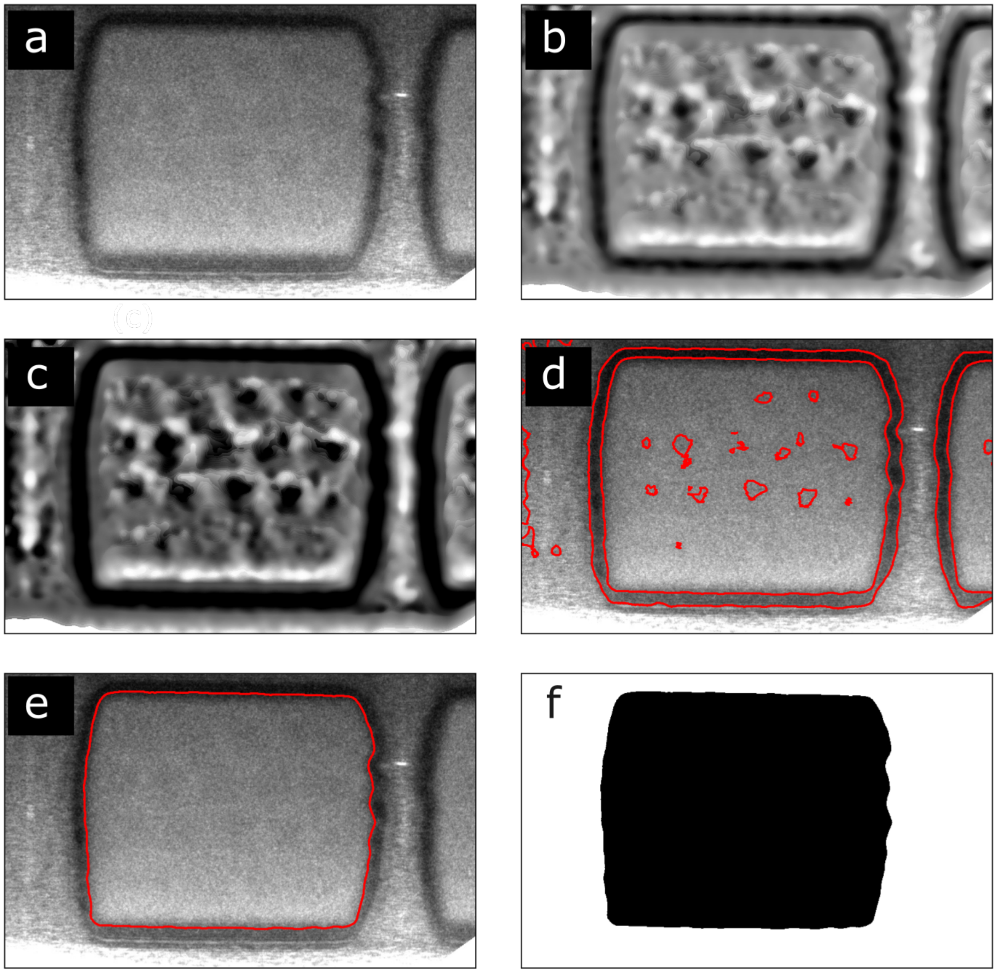

Figure S9: Mask generation process for simulation geometry (a) Bright field TEM image of block 1, (b) a gaussian blur is applied to smooth any sharp variations in intensity, (c) the image is limited in range to improve the accuracy of contour generation; the contours are shown in (d). (e) a single contour is selected to generate the mask (f) used for simulations.

References

(1) Richardson, M. The partial equilibrium diagram of of the FeGe system in the range 40-72 at. % Ge, and the crystallisation of some iron germanides by chemical transport reactions. *Acta Chemica Scandinavica* **1967**, *21*, 2305–2317.

(2) Egerton, R. F. *Electron Energy-Loss Spectroscopy in the Electron Microscope*, 3rd ed.; Springer: New York, 2011; Chapter 5.

(3) Ishitani, T.; Yaguchi, T. Cross-Sectional Sample Preparation by Focused Ion Beam: A Review of Ion-Sample Interaction. *Microscopy Research and Technique* **1996**, *35*, 320–333.

(4) Lipp, S.; Frey, L.; Lehrer, C.; Frank, B.; Demm, E.; Ryssel, H. Investigations on the topology of structures milled and etched by focused ion beams. *Journal of Vacuum Science and Technology B* **1996**, *14*, 3996–3999.

(5) Ziegler, J. F. `http://www.srim.org`.

(6) Wolf, D.; Schneider, S.; Rößler, U. K.; Kovàcs, A.; Schmidt, M.; Dunin-Borkowski, R. E.; Büchner, B.; Rellinghaus, B.; Lubk, A. Unveiling the three-dimensional magnetic texture of skyrmion tubes. *Nature Nanotechnology* **2021**,

(7) van der Walt, S.; Schönberger, J. L.; Nunez-Iglesias, J.; Boulogne, F.; Warner, J. D.; Yager, N.; Gouillart, E.; Yu, T. scikit-image: image processing in Python. *PeerJ* **2014**, *2*, e453.
